# Alopecia Universalis Occurring after Alemtuzumab Treatment for Multiple Sclerosis. A Two-Year Follow-Up of Two Patients

**DOI:** 10.3390/ijerph18147338

**Published:** 2021-07-09

**Authors:** Giovanna Borriello, Antonio Ianniello, Ahmed T Toosy

**Affiliations:** 1 MS Center, S. Andrea Hospital, Sapienza University of Rome, 00189 Rome, Italy; giovanna.borriello@gmail.com; 2 NMR Research Unit, Queen Square Multiple Sclerosis Centre, Department of Neuroinflammation, UCL Institute of Neurology, University College London (UCL), London WC1E 6BT, UK; a.toosy@ucl.ac.uk

**Keywords:** multiple sclerosis, Alopecia Universalis, Alemtuzumab, clinical neurology, safety, vitamin D, case report

## Abstract

Alopecia Universalis (AU) is the most severe form of Alopecia Areata and is caused by cytotoxic T-cells reacting with follicular autoantigens, producing complete loss of scalp and body hair. Alemtuzumab is a highly efficacious monoclonal antibody used in the treatment of Multiple Sclerosis (MS), but it causes secondary autoimmunity in up to 40% of patients. Many factors are believed to contribute to this process, but pathogenic mechanisms are not well clear. To date, three cases of AU after treatment with Alemtuzumab have been reported. In this paper we report the cases of two patients who developed AU 12 months after the second cycle of Alemtuzumab, with a review of the literature. One year after the end of the second cycle, two female patients in their thirties experienced complete hair loss. The first case was temporally associated with a significant drop in vitamin D (VD) levels. The second case was accompanied by joint swelling. Both patients had thyroid alterations and showed no hair regrowth after a 2-year follow-up. AU must be considered among the secondary autoimmune manifestations of Alemtuzumab treatment. We emphasize the need for appropriate patient screening and thorough clinical surveillance for factors predisposing patients to secondary autoimmunity.

## 1. Introduction

Alemtuzumab is a humanized monoclonal antibody that selectively targets CD52, a surface antigen expressed primarily on B and T lymphocytes and whose function is still largely unknown [1]. Two large Phase III trials and subsequent 9-year extensions have shown the sustained superiority of this drug on neurological disability, relapse rate and MRI outcomes, compared with interferon-beta 1 [2]. Recent real-world data confirmed the results of the registration studies [3]. However, the benefits of this therapy are counterbalanced by a significant incidence of adverse events, the most common of which regard secondary autoimmunity and affect up to 40% of patients. Alopecia Universalis (AU) is the most severe form of Alopecia Areata, an autoimmune condition directed at hair follicles. To our knowledge, only three cases of Alopecia Universalis (AU) after Alemtuzumab therapy for MS have been described so far [4,5,6]. Here we present the fourth and fifth cases of this kind, with a two-year-long follow-up. The two patients tried different topical therapies, but, so far, no hair regrowth has been noticed. Furthermore, we focus on the pathogenetic mechanisms that may lead to the development of this adverse effect of Alemtuzumab therapy.

## 2. Case Presentation

### 2.1. Case 1

A 32-year-old female patient presented with motor, sensory, and brainstem symptoms. Her MRI revealed enhancing and non-enhancing lesions in her brain and spinal cord and she was subsequently diagnosed with MS. She was previously healthy with no history of autoimmunity. She experienced clinical relapses and accumulation of new lesions under three different disease modifying therapies (DMTs), prompting escalation to Alemtuzumab. She received two courses of the treatment, the second (36 mg in total) in January 2018. Between the two courses the disease remained stable and the patient did not experience any adverse events.

**Figure 1:**
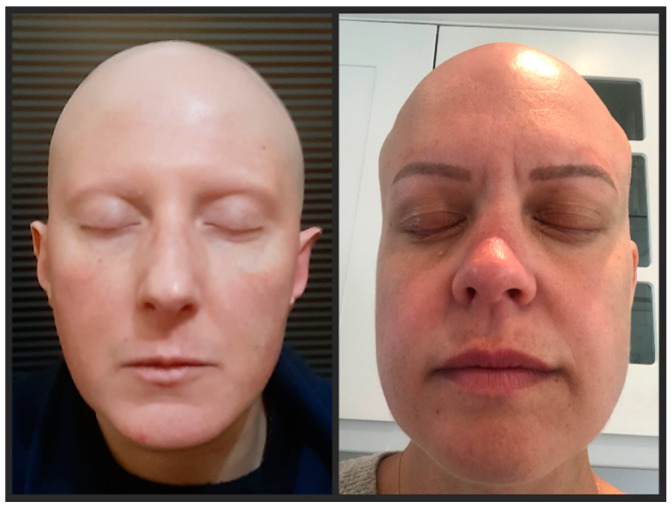
Pictures at 24 months after the onset of hair loss for patient 1 (**left**) and patient 2 (**right**, tattooed eyebrows).

Six months after the second course, she was diagnosed with Hashimoto’s thyroiditis and appropriate replacement therapy was commenced. In January 2019, the patient experienced patchy hair loss over her scalp which rapidly progressed over a month to involve the whole body, leading to a diagnosis of AU. The patient was treated with topical Minoxidil and Retinoic acid but, as of March 2021, no hair regrowth has been observed (Figure 1).

Notably, a decrease in VD concentration was noted prior to the onset of the AU (from 69 ng/mL in November 2018 to 8 ng/mL in January 2019), despite the patient already being on weekly supplementation. After increasing intake up to 5000 IU/day, serum VD levels normalized. The most recent 3T MRI scan in December 2020 showed stable lesion load and no enhancements.

### 2.2. Case 2

A 36-year-old female with an unremarkable past medical history was diagnosed with MS in 2015 after a right optic neuritis and a spinal relapse. She experienced gastrointestinal side effects with Beta-interferon 1 treatment, and was switched to Alemtuzumab. Two courses of Alemtuzumab were administered in October 2016 and 2017. Her MS remained inactive. Of note, TSH levels were slightly raised (5.98 mIU/L; reference: 0.27–4.20) on several occasions after treatment onset but returned to normal without replacement therapy. The patient had begun VD supplementation soon after the diagnosis, but never followed a precise scheme and her levels were never tested.

In October 2018, the patient presented with non-scarring patchy hair loss all over her body, which spread over three months into a universal pattern. Concomitantly, she experienced painless finger joint swelling in her hands. She was empirically treated with intravenous methylprednisolone (1000 mg for 3 days followed by oral tapering), with resolution of articular symptoms. ANA, Rheumatoid Factor, and Anti-CCP produced negative results. Topical therapy with Pimecrolimus and Beclomethasone was also tried, but after 26 months no hair regrowth has occurred (Figure 1). The most recent 3T brain MRI in December 2020 showed lesion stability for her MS.

## 3. Discussion

Alemtuzumab is a humanized monoclonal IgG1 antibody that induces a rapid and long-lasting depletion of CD52 expressing cells, mostly B and T-lymphocytes. The subsequent repopulation from precursors follows a temporal pattern that varies greatly amongst different cell populations: CD4+ and CD8+ T lymphocytes return within normal values after, respectively, 60 and 31 months, while B cells not only complete repopulation in 3 months, but also show a hyper-repopulation up to 165% of baseline values at 12 months [1]. The long-lasting depletion of CD4+ cells may account for the sustained efficacy of the drug, even without continuous treatment [7].

Nevertheless, secondary autoimmunity is reported as a common adverse event of this DMT and affects 30–40% of patients. Secondary autoimmune diseases usually involve thyroid, kidney, and platelet function [7,8]. Recently, new evidence has emerged regarding serious, in some cases fatal, adverse events, including cardiovascular disorders.

Multiple factors are believed to lead to the development of secondary autoimmunity. The unregulated B cell pool expansion, together with the increase in B cell activating factor (BAFF) concentration have been associated with uncontrolled antibody production in response to self-antigens [8,9].

Another proposed mechanism is homeostatic peripheral expansion (HPE) [10]. When lymphopenia occurs, peripheral T cells that escaped depletion are stimulated to proliferate in response to both low-affinity self and non-self-antigens, in order to restore the T repertoire. When this “first hit” is coupled with a “second hit”, such as the over-expression of pro-inflammatory cytokines, reduced thymic output, and the Treg/non-Treg ratio skewing, given a genetic predisposition to autoimmunity, self-tolerance is broken.

Secondary autoimmune diseases appear 6–61 months after the first infusion of Alemtuzumab, but usually peak during the third year after the initiation of the therapy, consistent with the reconstitution kinetics of the T-cell population [1].

AU causes complete non-scarring loss of both scalp and body hair, and is caused by cytotoxic T-cells reacting with follicular autoantigens. Although several studies have shown some degree of hair regrowth with different drugs, currently no completely effective treatment has been approved for this condition [11].

Serum VD levels are lower in patients with AA than healthy subjects and inversely correlate with disease severity [12]. This is related to the well-known effects of VD, both on the hair follicle and on the immune system. In particular, VD levels are negatively associated with IL-21 and IL-17 concentrations [13]. In turn, these proinflammatory cytokines have been shown to induce Th17 cells and inhibit the re-differentiation of regulatory T cells, playing an important role in the development of many autoimmune diseases. In particular, IL-21 levels are directly related to the development of autoimmune diseases after Alemtuzumab administration in MS [14].

Severe subtypes of AA are also associated with increased levels of anti-thyroid antibodies [15]. Furthermore, autoimmune alopecia and thyroid autoimmunity share the same T cell mediated pathogenesis [16]. Both conditions are associated with severe VD deficiency, thus highlighting need for VD supplementation in patients undergoing immunomodulatory treatments.

Although secondary autoimmune diseases after Alemtuzumab treatment are extensively reported in the literature, alopecia is considered only a rare occurrence [17]. A literature review showed only six reports of AA after Alemtuzumab [4,18], while Alopecia Universalis has been described only three times so far.

The first two cases of AU following Alemtuzumab occurred six months after the second cycle and progressed over three months [5,6]. Both patients had no history of autoimmune disease, nor positivity for anti-thyroid antibodies. Both declined therapy for the condition. Nevertheless, the first patient did not experience any hair regrowth over six months of follow-up, whilst the second patient experienced complete hair regrowth, two years after the last infusion.

The third report of AU associated with Alemtuzumab in MS documented hair loss five months after the second infusion and was accompanied by worsening of pre-existing vitiligo, a skin disease often associated with autoimmune alopecia [4].

None of the three previous cases had autoimmune thyroid dysfunction. Moreover, none of the reports mentioned VD levels, a feature that would be interesting to examine, as the hair loss in our first patient was concurrent with a relevant decrease in VD concentration.

We report two further cases of AU after Alemtuzumab, with the longest time following last drug administration and with the longest follow-up after the onset of the adverse event. It is possible that decreases in VD may predispose patients to AU post-Alemtuzumab in the context of an autoimmune environment (supported by the findings of thyroid antibodies and small joint swelling in our cases), although future studies could investigate this proposition.

## 4. Conclusions

AU is a rare autoimmune complication of Alemtuzumab treatment in MS, occurring 5–12 months after the second cycle, consistent with the most typical timing of appearance of secondary autoimmune diseases. Its pathogenesis, albeit not entirely understood, is T-cell related and seems linked to the homeostatic peripheral expansion phenomenon.

These cases show that autoimmune alopecia should be considered among the possible adverse events of the therapy with Alemtuzumab and highlights the need to screen patients for possible factors predisposing them to secondary autoimmunity before and after treatment.

## Data Availability

The data presented in this study are available on request from the corresponding author.
